# Clinical efficacy of phentolamine in the treatment of feeding intolerance in premature infants with low birth weight

**DOI:** 10.12669/pjms.36.7.2633

**Published:** 2020

**Authors:** Hongya Li, Bin Li, Xuehua Wen

**Affiliations:** 1 Hongya Li, Department of Neonatology, The First Central Hospital of Baoding, Baoding, Hebei, 071000, P.R. China; 2 Bin Li, Department of Neonatology, Maternal and Child Care Service Centre of Baoding, Baoding, Hebei, 071000, P.R. China; 3 Xuehua Wen, Department of Neonatology, The First Central Hospital of Baoding, Baoding, Hebei, 071000, P.R. China

**Keywords:** Phentolamine, Feeding intolerance, Low birth weight infants, Premature infants

## Abstract

**Objective:**

To discuss the clinical efficacy of phentolamine in the treatment of feeding intolerance in premature infants with low birth weight.

**Methods:**

Seventy-one low-birth-weight infants with feeding intolerance were randomly divided into the phentolamine group and the erythromycin group (38 patients and 33 patients, respectively). The infants were given basic treatment, such as gastric lavage, temporary fasting, nutritional support and abdominal massage. The phentolamine group was intravenously pumped with phentolamine as the basis of basic treatment, while the erythromycin group was given erythromycin as the basis of basic treatment. The time for gastrointestinal symptoms to disappear, the time the basic standard was reached, the time parenteral nutrition was used, the total time enteral feeding was implemented, the length of stay, and the increase in physical indexes according to the corrected gestational age of 40 weeks of the two groups were compared.

**Results:**

There was no significant difference between the phentolamine group and the erythromycin group in vomiting disappearance time or the increase in physical indicators at the corrected gestational age of 40 weeks (P>0.05), while the abdominal distension disappearance time, the time of restoration to birth weight, the time to reach the basic standard, the total time of parenteral nutrition, the total time of enteral feeding, and the length of stay in the phentolamine group were shorter than those in the erythromycin group, with significant differences (P<0.05).

**Conclusion:**

Phentolamine has a significant effect on alleviating symptoms and shortening the treatment time while treating feeding intolerance in premature infants with low birth weight, without adverse events, so it is worthy of clinical promotion.

## INTRODUCTION

Feeding intolerance (FI) refers to a group of clinical syndromes of intolerance to enteral nutrition caused by gastrointestinal disturbance of newborns, but its etiology is currently still unclear. Due to the immature development of various nerve reflexes and the influence of gastrointestinal and swallowing dysfunction, most premature infants have varying degrees of feeding intolerance, which affects their growth and development as well as quality of life.^1^ There is a high incidence of FI in premature infants, which may develop into life-threatening neonatal necrotizing enterocolitis (NEC)^2^ and lead to poor psychological development.^3^ The conversion of low-birth-weight premature infants from tube-feeding nutrition to full oral milk feeding is one of the criteria for premature infants to be discharged from the hospital.^4^ Therefore, it has become a common concern of the medical staff in the neonatal intensive care unit to promote the safe and effective oral feeding of premature infants as soon as possible. The intravenous infusion of phentolamine was used in this study to treat feeding intolerance in premature infants, and good clinical efficacy was obtained. The experience is summarized as follows.

## METHODS

Seventy-one low-birth-weight premature infants aged less than 1.0 day who were admitted to the Neonatology Department of Maternal and Child Care Service Center of Baoding and our hospital from January 2015 to July 2018 were enrolled in this study.

### Inclusion criteria

1500 g ≤ birth weight < 2500 g, 224 d (32 weeks) ≤ gestational age < 258 d (37 weeks). The patients were randomly divided into the phentolamine group and erythromycin group (38 patients and 33 patients, respectively). There was no significant difference between the two groups in sex, gestational age or body weight before treatment (P >0.05; Table-I).

**Table-I:** Comparison of general information between the two groups before treatment.

Group	Sex (male/female)	Body weight (g)	Gestational age (d)
Phentolamine group	20/18	2032±258	246±8.5
Erythromycin group	17/16	2080±338	242±8.9

### Ethical Approval

The study was approved by the Institutional Ethics Committee of Baoding First Hospital, and written informed consent was obtained from all participants.

### Diagnostic Criteria

First, surgical conditions, such as congenital gastrointestinal malformation, necrotizing enterocolitis and normal gastrointestinal feeding, were excluded. The diagnostic criteria were as follows: (1) vomiting refers to vomiting ≥3 times per day; (2) abdominal distension refers to a 24-h abdominal circumference increase of ≥1.5 cm, accompanied by visible peristalsis; (3) gastric retention refers to the storage of more than 1/3 of previous milk or coffee-ground or bile-like substances in the stomach.

### Therapeutic methods

After admission to the hospital, both groups of children were kept warm and given total intravenous alimentation, anti-infection treatment and other supportive treatment to ensure water, electrolyte and acid-base balance; when necessary, the intravenous infusion of plasma and gamma globulin was performed for immunoenhancement, and CPAP and mechanical ventilation therapy were used for respiratory failure. All treatment methods were used in strict accordance with the rules of diagnosis and treatment in the *Guidelines for the Management of Premature Infants:* After the children developed feeding intolerance, they were given gastric lavage, temporary milk ban, abdominal massage, supplementation of intestinal probiotics and other basic treatments. The phentolamine group was given an intravenous infusion of phentolamine as the basis of basic treatment. The liquid configuration method was used: phentolamine (1.8 mg/kg) was added to a 10-ml 5% glucose injection for 10 h of continuous pumping once/day with a pumping speed of 0.3 μg/(kg·min) for 3-5 days. The erythromycin group was given erythromycin, 5 mg/kg, once daily as the basis of basic treatment, which was added to the 20-ml 10% glucose injection for 10 hour continuous pumping (1 mg/(kg·h), once daily, 3-5 days). The intravenous nutrition support for both groups was discontinued when total enteral nutrition was performed. During the study, daily changes in the infants were recorded, including basic vital signs, daily milk-taking volume, residual milk volume, daily weight changes, and symptoms of vomiting or abdominal distension. The following indexes were compared: (1) time of disappearance of gastrointestinal symptoms (vomiting and abdominal distension); (2) time to reach the basic standard [the time required for the amount of milk fed orally after birth to reach 100 ml/(kg•d)]; (3) the time of total parenteral nutrition (TPN); (4) the time to reach total enteral nutrition (TEN); (5) the length of stay; and (6) the increase in physical indexes (body mass, body length and head circumference) at the corrected gestational age of 40 weeks.

### Statistical Analysis

The SPSS 19.0 statistical software package was used for analysis, with the measurement data with a normal distribution expressed as the mean ± standard deviation (x(-)±s), and the intergroup comparison was performed by the independent sample t test. P < 0.05 was considered statistically significant.

## RESULTS

The time to disappearance of vomiting between the phentolamine treatment group and the erythromycin group showed no significant difference (P >0.05), but the disappearance time of abdominal distension, the time of recovery to birth weight, the length of stay, the time to reach basic standard, and the time of TPN and TEN showed statistically significant differences in the erythromycin group (P < 0.05; Table-II). There was no significant difference in body weight, body length or head circumference between the two groups at the corrected gestational age of 40 weeks (P >0.05; Table-III).

**Table-II:** Comparison of indexes between the two groups after treatment (*x̄*±s).

Group	Disappearance of vomiting (d)	Disappearance of abdominal distension (d) (d)	Recovery to birth weight (d)	Length of stay (d)	Reached the basic standard (d)	Time of TPN (d)	Time of TEN (d)
Phentolamine group	4.0±1.8	3.2±1.3*	6.8±1.5*	13.3±3.9*	10.9±3.4*	11.6±3.8*	12.9±3.6*
Erythromycin group	3.7±2.0	3.2±1.9	8.6±1.8	20.7±9.6	17.6±11.1	17.6±11.6	22.1±12.5
T Value	0.28	5.15	4.06	50.1	24.3	29.1	42,8
P	P>0.05	P<0.05	P<0.05	P<0.05	P<0.05	P<0.05	P<0.05

* Note: compared with the erythromycin group, P<0.05

**Table-III:** Comparison of the increase in physical indexes between the two groups at the corrected gestational age of 40 weeks (*x̄*±s).

Group	Cases	Body weight (g)	Body length (cm)	Head circumference (d)
Phentolamine group	38	4.2±1.0	3.4±0.9	4.1±0.9
Erythromycin group	33	3.2±1.1	2.6±1.0	3.0±1.1
T value		0.01	3.67	1.59
p		P>0.05	P>0.05	P>0.05

## DISCUSSION

In China, the rate of premature infants is high and is exhibiting an obvious increasing trend as the number of second births of elderly parturient women and in vitro fertilization pregnancies increases.^5^ The majority of premature infants are low-birth-weight, very-low-birth-weight or extremely low-birth-weight infants. Low-birth-weight infants are relatively mature in the development of various systems, and feeding is the prominent problem of premature infants at this stage. Therefore, the feeding intolerance of low-birth-weight premature infants was selected as the topic for this study.

The central nervous system of premature infants is not fully developed, and its regulation of the oral cavity, throat and airway is immature; therefore, parenteral nutrition is generally needed to assist enteral nutrition for preterm infants. Feeding intolerance is a common clinical complication among premature infants, which may cause gastrointestinal bleeding and necrotizing enterocolitis if severe. The length of stay will be longer, and long-term intravenous hyperalimentation is required if there is no effective treatment, which may easily lead to complications, such as cholestasis, bleeding and infection.^6^ Therefore, it is very important to adopt active treatment methods to reduce feeding intolerance.

The non-drug clinical treatment methods for feeding intolerance in premature infants usually include newborn touch, kangaroo nursing, non-nutritive sucking, establishment of a breast milk bank, application of deeply hydrolyzed milk powder, supplementation of intestinal probiotics, etc., and all of these methods have achieved good efficacy.^7^-^9^ As a kind of drug therapy, erythromycin has a pro-kinetic effect on the full digestive tract, which can enhance esophageal contraction, increase the pressure on the lower esophageal sphincter, enhance gastric antrum contraction and gallbladder contraction and promote colon movement.^10^ Low-dose erythromycin is usually used to treat neonatal feeding intolerance, which can activate the gastrointestinal motility receptor to cause mild contraction of the digestive tract and improve feeding tolerance, thus shortening the time of parenteral nutrition and achieving early enteral feeding. In addition, the intravenous administration of erythromycin in the treatment of feeding intolerance in premature infants has been suggested in many studies^11^ and is widely applied in clinical work.

Nevertheless, the treatment of feeding intolerance in premature infants with low-dose erythromycin is the only drug option, so it is necessary to further explore a variety of drug treatment methods to achieve individualized treatment and address feeding intolerance in premature infants at different gestational ages.

Phentolamine, an α-receptor blocker, can competitively block the binding of norepinephrine to the α receptor, thus relieving vasoconstriction. Moreover, phentolamine can directly dilate arterioles and bronchi and improve pulmonary ventilation function to improve systemic microcirculation and oxygen supply.^12^ In the neonatal stage, phentolamine can be applied externally to improve microcirculation.^13^ In recent years, there have been increasing reports on the treatment of neonatal persistent pulmonary hypertension with phentolamine^14^, but there are few Chinese and international reports on the treatment of feeding intolerance with phentolamine.

Improving the intestinal blood supply is the key to the treatment of feeding intolerance in premature infants. Phentolamine could block the binding of norepinephrine to the α receptor and relieve the vasoconstriction effect to improve the blood circulation of the whole body of children, thus achieving the effect of improving intestinal microcirculation. After the intestinal microcirculation is improved, the congestion and edema of the intestinal wall can be alleviated. Moreover, phentolamine could antagonize the relaxation effect of epinephrine on gastrointestinal smooth muscle and enhance intestinal peristalsis, thus eliminating the intestinal accumulation of gas, restoring intestinal movement and absorption function, and significantly improving abdominal distension and other conditions.

According to the results of this clinical experiment, compared with the erythromycin group, the phentolamine group could shorten the duration of abdominal distension in premature infants with low birth weight, increase the intake of milk, reduce the time of intravenous nutrition, and shorten the length of stay (P<0.05. As such phentolamine could effectively treat feeding intolerance in premature infants with low birth weight. Due to the strict control of the amount of phentolamine in the body per unit time, no adverse reactions, such as nasal obstruction, rash, flushed face and arrhythmia, etc. were observed in the phentolamine group.

## CONCLUSION

Phentolamine has a good therapeutic effect on the treatment of feeding intolerance in premature infants with low birth weight. This method is simple and practical, is not associated with adverse reactions and is worthy of further clinical promotion.

### Authors’ Contributions:

**HL &** **BL:** designed this study and prepared this manuscript.

**XW:** Collected and analyzed clinical data, also revised this manuscript.
